# Dynamic changes in epithelial cell morphology control thymic organ size during atrophy and regeneration

**DOI:** 10.1038/s41467-019-11879-2

**Published:** 2019-09-27

**Authors:** Thomas Venables, Ann V. Griffith, Alice DeAraujo, Howard T. Petrie

**Affiliations:** 1 The Scripps Research Institute, 130 Scripps Way, Jupiter, FL 33458 USA; 2 Present Address: Department of Microbiology, Immunology and Molecular Genetics, UT Health San Antonio, San Antonio, TX 78229 USA

**Keywords:** Imaging, Lymphocyte differentiation, Imaging the immune system, Thymus

## Abstract

T lymphocytes must be produced throughout life, yet the thymus, where T lymphocytes are made, exhibits accelerated atrophy with age. Even in advanced atrophy, however, the thymus remains plastic, and can be regenerated by appropriate stimuli. Logically, thymic atrophy is thought to reflect senescent cell death, while regeneration requires proliferation of stem or progenitor cells, although evidence is scarce. Here we use conditional reporters to show that accelerated thymic atrophy reflects contraction of complex cell projections unique to cortical epithelial cells, while regeneration requires their regrowth. Both atrophy and regeneration are independent of changes in epithelial cell number, suggesting that the size of the thymus is regulated primarily by rate-limiting morphological changes in cortical stroma, rather than by their cell death or proliferation. Our data also suggest that cortical epithelial morphology is under the control of medullary stromal signals, revealing a previously unrecognized endocrine-paracrine signaling axis in the thymus.

## Abstract (web summary)

One aspect of ageing on immunity is attributed to accelerated thymic atrophy, but the underlying mechanism is still lacking. Here the authors show, using conditional reporter mouse models, that both atrophy and regeneration of the thymus are regulated by rate-limiting morphological changes in epithelial stroma, independent of cell death or proliferation.

## Introduction

Like all blood cells, T lymphocytes are lost throughout life, and must be continuously replaced. The thymus is the primary site of post-natal T lymphopoiesis. However, unlike other tissues that undergo steady-state differentiation, there are no lymphoid stem cells contained within the thymus, and instead it depends on recruitment of multipotent progenitors from the blood^1^. Upon entering the thymus, microenvironmental conditions unique to the organ specify T lineage commitment, as well as proliferation, tolerance, T lineage divergence, and other processes. Ultimately, new T lymphocytes are exported to the periphery. Since the hematolymphoid components of the thymus are quite transient, it is obvious that they cannot define its durable identity. Instead, this identity is established by the stromal components of the thymus, primarily including specialized thymic epithelial cells (TEC, reviewed in refs. ^2,3^). TEC provide most of the signals that induce and control T lymphocyte production (reviewed in ref. ^4^). Reciprocally, they depend on the presence of lymphocytes for their differentiation and organization^5–7^, a process referred to as crosstalk. Cortical TEC (cTEC) also establish the physical matrix for directional migration of lymphoid progenitor cells within the thymus^8,9^, simultaneously limiting thymus size by providing a rate limiting number of progenitor niches in this matrix^10^. Medullary TEC (mTEC) are essential in imposing self-tolerance in new T cells prior to export to the periphery^11^, and are likely to play roles in T lineage divergence and functional maturation as well. Thus, epithelial cells exhibit diverse essential roles in maintaining T cell production and function.

In other tissues, epithelial cells exhibit distinctive squamous, cuboidal, or columnar morphologies, and are polarized (with respect to a basement membrane) in either a single simple layer or in multiple stratified layers^12^. In the thymus, with the exception of a small proportion of conventional epithelial cells lining the capsule and blood vessels, most TEC lack classical epithelial morphology^13–16^. Instead, they are defined as epithelial mainly based on biochemical features, such as the appearance of desmosomes or keratin filaments^13,15,17^. Various histochemical markers indicate that cTEC, in particular, form an extensive network of finely branched cell processes^14,15,17^, but the morphology and number of individual cells in this network has been very difficult to define, due to this elaborate branching morphology and their relatively uniform staining with various antibody markers. Medullary TEC (mTEC) appear to be less dense, and therefore more easily defined as individual cells, but extensive heterogeneity among lineage markers has rendered the morphology of individual mTEC vague as well. Consequently, defining the size, shape, and interconnectivity of these essential cells has remained enigmatic.

Given the need for continuous T cell production during life, the thymus is paradoxically the most rapidly aging tissue in the body. It reaches peak tissue mass (in all species studied) prior to the onset of adolescence, and exhibits rapid and progressive atrophy afterwards, such that by mid-life most healthy mass is lost^18,19^. Except at very late age^20^, thymic lymphocytes are essentially unchanged in the atrophied thymus, while these age related changes are primarily manifest in stromal cells, particularly cortical^21^. As mentioned above, niche availability provided by cTEC is the rate limiting feature for lymphoid cellularity and thymus size^10^. Thus, as cTEC deteriorate during aging, the thymus becomes proportionally smaller. Since new T cells are produced proportionally to thymic mass^22,23^, peripheral homeostasis thus becomes more dependent on homeostatic expansion of existing T cells, with the repertoire gradually drifting towards immunologic memory, with diminished broad spectrum immunity as a result^24^.

Remarkably, the atrophied thymus retains potent regenerative capacity, and can be induced to attain its full peak size^25–27^ by stimuli such as androgen ablation. Quite logically, albeit without much evidence, thymic atrophy is assumed to result from senescence-associated cell death among TEC, while regeneration is believed to result from proliferative expansion from an epithelial stem or progenitor cell population. Consistent with these concepts, experimental loss of cTEC does result in decreased thymus size^28^, while induction of cTEC proliferation results in a larger thymus^29^. However, the fact that thymus size changes in response to TEC number (and resulting lymphoid capacity) does not mean that atrophy or regeneration, under physiologic conditions, necessarily involve changes in TEC number.

The present study stems from a large temporal database of stromal transcriptional profiles during aging and regeneration, as described later in this manuscript. Non-presumptive analysis indicates that dynamic changes in genes associated with cell size and cell morphology dominated the regeneration response. Here we use two different conditional reporter models to morphologically define and enumerate TEC in young, aged, or regenerated thymuses. Both models confirm the predictions of informatic analysis, showing that age atrophy of the thymus represents contraction of unique cell projections that characterize cTEC, while regeneration involves their regrowth. Both atrophy and regeneration occur independently of changes in cTEC number, and appear to solely reflect changes in projection morphology. Although mTEC morphology does not change appreciably with age, dynamic analysis suggests that medullary stroma may play an important role in modulation of cTEC morphology via paracrine production of known morphogens and growth factors. Our findings reconcile diverse existing concepts, and provide a revised view of atrophy and regeneration based on structural remodeling of a novel cTEC morphology that is unique among metazoan tissues.

## Results

### Changes in cell projection genes dominate regeneration

**Fig. 1:**
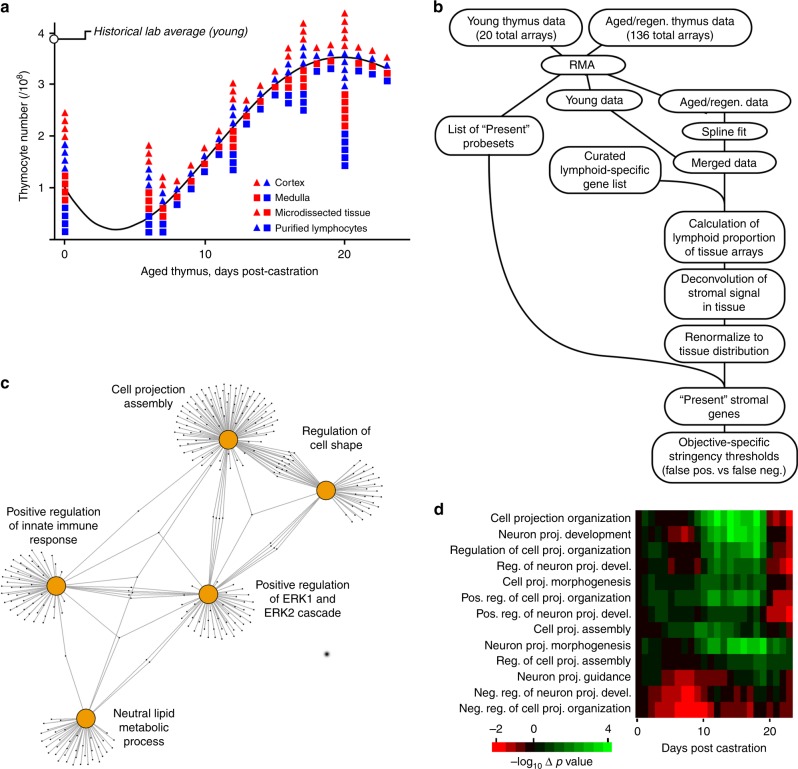
Changes in cell morphology genes dominate the regeneration response. **a** Summary of 136 microarrays collected during castration-induced regrowth of the age atrophied thymus, mapped onto a regression curve of organ size over time (see text). An additional 20 microarrays from young thymus, 5 for each cell/tissue type, are not indicated (because they are off the time scale), representing a total of 156 microarrays used for this study. Each tissue:lymphoid pair also provides a deconvolved stromal signature (78 total) used for subsequent analysis. **b** General workflow of steps in the deconvolution of stromal gene expression. **c** A network map of the top 5 GO:BP categories (200 genes or fewer) indicates a strong bias in cortical stroma towards dynamically regulated genes associated with cell projection morphology. Connections between hubs indicate shared genes. **d** Mapping of deconvolved thymic stromal transcriptomes onto GO categories related to cell projection (and containing at least 10 total genes). The heat map shows the change in statistical significance (i.e., change in Fisher exact test *p* value) over time of regeneration, normalized to day 0. There is a negative enrichment of negative regulators early, and a positive enrichment of positive regulators late, further supporting the prediction from non-presumptive analysis that changes in cell shape may play a significant role in the regeneration response

While thymic lymphocytes have been exhaustively studied, stromal elements that control their production have been obscured by difficulties in their isolation (the reasons for which will be made even more clear later). To circumvent this, we implemented a signal deconvolution algorithm to derive stromal transcriptomes from microdissected thymic tissue and the corresponding (purified) lymphoid components. A total of 156 microarrays are included in the present study, representing 39 medullary and 39 cortical tissue samples, and 39 medullary and 39 cortical lymphoid samples, from 5-week-old (young) mice, or 12-month-old (aged) mice before, or at various points after, castration (Fig. 1a). Regeneration data were fit to a 3rd degree polynomial spline, and in it’s simplest representation, the tissue:lymphoid ratio at any point in time can be used to deconvolve the corresponding stromal transcriptome (see Fig. 1b and Methods section), thus resulting in 78 global stromal transcriptomes over time, half in each tissue compartment. For convenience, the union of stromal genes expressed at any point during the regeneration process is shown in Supplementary Data 1.

We initially focused on dynamic changes in cTEC, since this compartment dominates both atrophy and the regeneration response^21,30–32^. When dynamically regulated stromal genes were mapped onto Gene Ontology (GO)^33^ Biological Process (BP) categories (Fig. 1c), two of the five most statistically significant (i.e., over-represented) categories (empirically limited to categories with 200 genes or fewer, since very large categories always tend to be significant) were explicitly involved in cell morphology (regulation of cell shape and cell projection assembly). Two of the remaining three (positive regulation of ERK cascade and neutral lipid metabolic process) are also implicated in cell morphology and cell membrane projections, just not as exclusively as the others. Not surprisingly, then, dynamically regulated genes in the stromal response (Supplementary Data 1) are extensively populated by genes associated with cell adhesion, guidance response, membrane proximal signaling, and cytoskeleton remodeling. These observations, together with the known branching morphology of TEC (especially cTEC), prompted us to look further into this enrichment. GO BP ontologies containing the term projection, and the genes associated with them, were identified, and those ontologies containing at least 10 members were extracted. For each, over-representation of genes in the stromal list at each time point was tested using the Fisher exact test (corrected for false discovery rate, all genes as background). The results are shown in Fig. 1d, normalized to day 0 (12 months of age, pre-castration). Remarkably, all projection BP exhibited significant regulation of cell projection genes, with two main patterns found, namely, depletion of negative regulators early followed by restoration later, or enrichment of positive regulators early followed by their depletion later. These patterns align closely with the regeneration kinetics we have previously described (Fig. 1a, and ref. ^21^), and prompted us to speculate that changes in stromal morphology, rather than changes in stromal cell number, might explain thymic atrophy and regeneration.

### Genetic labeling reveals a unique morphology for cTEC

**Fig. 2:**
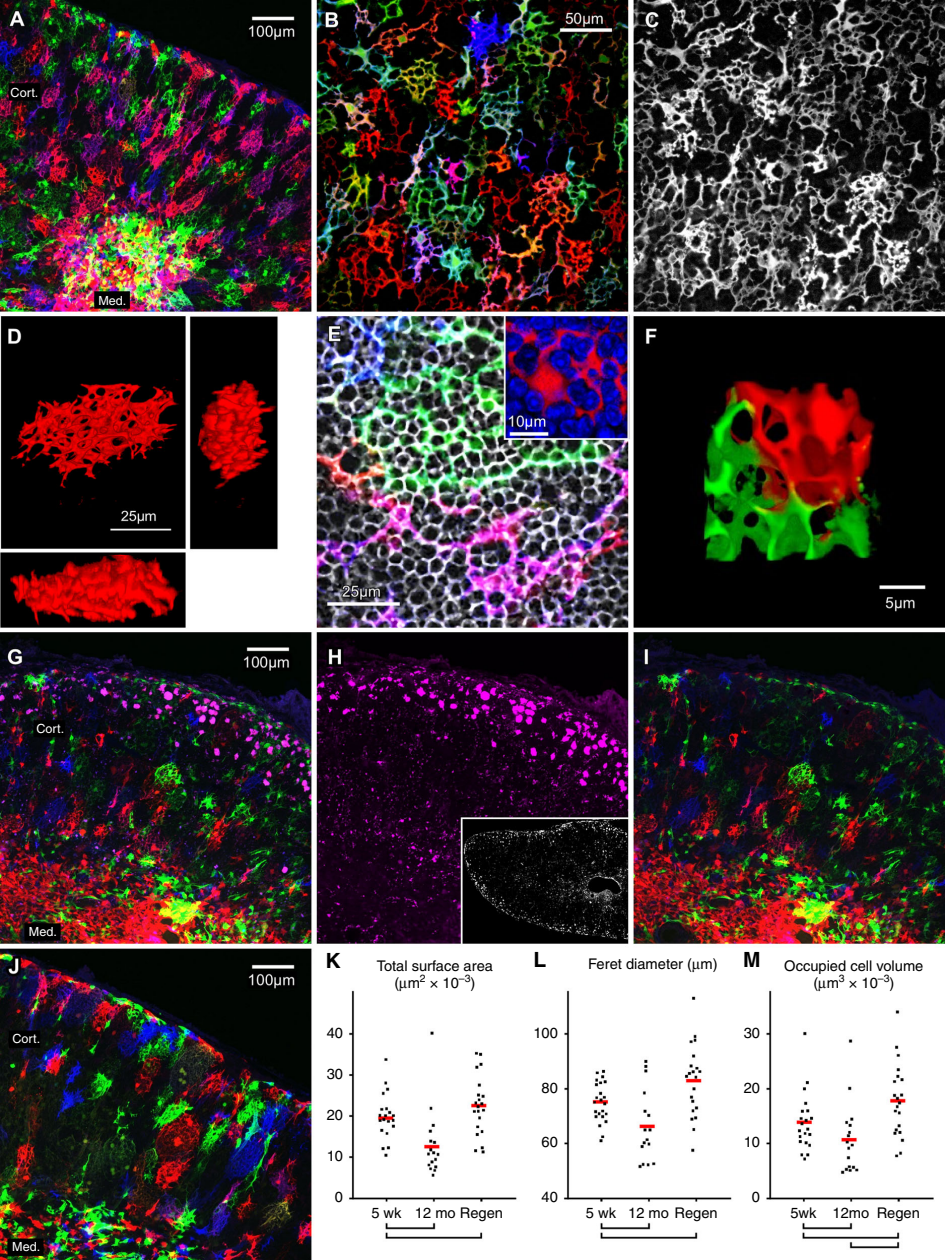
cTEC exhibit a unique morphology that fluctuates during aging and regeneration. Data are from *Foxn1[Cre]*^*+*^ *Rosa26[Confetti]*^*+*^ thymuses. Blue = Cfp, green = Yfp, red = Rfp. Mouse age was: **a**–**f**, 5 weeks, **g**–**i**, 12 months, and **j** is peak regenerated. Comparable panels (e.g., **a**, **i**, **j**) were processed identically. 3D renderings can be found in Supplementary Movies 2–4. **a** Maximum Z projection of a 40 µm optical stack (the approximate transverse depth of a single cTEC) at 5 weeks. **b** Zoomed view of the cortex (6 µm Z projection), revealing distinctions between individual cells, in contrast to **c**, which is conventional cTEC marker expression (Krt8) in the same stack. Scale bar in **b** also applies to **c**. **d** Orthogonal views of a typical cTEC, revealing looping structures forming a complex labyrinth of intracellular voids filled by lymphoid cells (**e**), as indicated by Thy-1 staining (gray) or DAPI staining (inset, blue). **f** 2D representation of the interface between two cTEC, showing that most contact occurs via abutment of looping structures, rather than synapses. **g** Maximum Z projection of a 40 µm-thick optical stack from a 12-month-old mouse. Confetti detection is obscured by autofluorescent pigments associated with aging, but these can be imaged independently (**h**), and thus subtracted (**i**; see also Supplementary Fig. 2). Scale bar in **g** also applies to **h** and **i**. Inset in **h** is at 20× magnification. cTEC in the aged thymus (**i**) are much less conspicuous than in young (**a**), reflecting both contraction of total cell dimensions (**k**–**m**) and thinning of the remaining projections. **j** 40 µm Z projection from a 12-month-old mouse on the peak of regeneration (day 20). Confetti intensity is much more robust than in the aged thymus (**j**), but is not restored to the status of the young (**a**), with some cells retaining an aged phenotype (atrophic), while others are abnormally large. **k**–**m** measurement of relevant physical parameters in cTEC, such as total surface area, feret diameter, or cell volume. Brackets indicate statistical significance (two-tailed *t* test, *p* value < 0.05). *n* = 3 biologically independent tissues each for 5 weeks, 12 months, and regenerated groups. Source data are provided in the accompanying Source Data File

Based on standard immune marker staining, cTEC are known to possess extensively branched cell processes^14,15,17^ but conventional staining cannot distinguish where one cell ends and the next begins, or the size or shape of individual cells. To fill this void, we utilized the Confetti (Brainbow2.1) conditional allele^34^, wherein the *Rosa26* locus has been modified to include a floxed stop codon upstream of two cassettes of two fluorescent proteins in inverted transcriptional orientation, with each cassette flanked by inverted loxP sites. In the presence of Cre, the stop codon is deleted and one of the four fluorescent proteins is placed under the transcriptional control of *Rosa26*. The four fluorescent proteins are a membrane-targeted cyan fluorescent protein (mCfp), a nuclear targeted green fluorescent protein (nGfp), and cytoplasmically retained yellow and red fluorescent proteins (cYfp, cRfp). nGfp is not useful for determining TEC morphology, since it labels only the nucleus. Reporter activation in virtually all TEC (see Supplementary Fig. 1) was induced via an allele containing a bicistronic Cre knockin of the *Foxn1* locus^35^. Images from such thymuses are shown in Fig. 2. A wide area view (50 μm maximum Z projection) of tissue from a young adult mouse thymus (5 weeks) is shown in Fig. 2a, revealing randomly colored TEC in a variety of hues (note: as long as Cre is expressed, the Confetti allele can continue to rearrange, and thus intermediate mixed hues can be seen). A 3D axial video of this same projection volume is shown in Supplementary Movie 1. A higher magnification of a cortical region is shown in Fig. 2b. The ability of the Confetti allele to define individual cells where conventional cTEC markers cannot (in this case, keratin-8) is highlighted by Fig. 2c. Viewed in 3D (see orthogonal projections in Fig. 2d, or the associated animation in Supplementary Movie 2), individual cTEC exhibit a morphology not seen in any other cell or tissue type. Consistent with conventional immunostaining, they exhibit an extensive network of projections. However, these projections are not branches in the traditional sense, i.e., progressively thinning bifurcations resulting in terminal ends, like a neuron or dendritic cell. Instead, cTEC cell projections form of long, looping structures that, in turn, generate an extensive intracellular labyrinth of 20–30 voids in each cTEC. Each void, in turn, contains approximately 2–10 cortical lymphoid cells (as determined by DAPI or CD90 staining, see Fig. 2e), totaling about 100–150 lymphoid cells almost totally engulfed by each cTEC. Connections between adjacent cTEC occur mainly through abutment of opposing loops (Fig. 2f, and Supplementary Movie 3), rather than via synapsed terminal ends (as in neurons, for instance), although some connections via synapses are also seen. Although the fine structure of individual loops is variable, overall shape, size, and orientation in cTEC is fairly consistent (best visualized with young thymus in Supplementary Movie 1), representing a slightly flattened ovoid (see Fig. 2d) approximately 75 ± 10 μm in its long dimension, which is radially aligned towards the capsule. Long axis radial alignment in cTEC, was first proposed over 30 years ago^15^, and shows that like other epithelial types, cTEC are polarized, although it mostly obscured by their complex morphology and deep embedding in lymphocytes.

In the 12-month-old thymus, which is about 25% of the volume/cellularity found in the young thymus^21^, dramatic differences are seen (Fig. 2g–i). Pigment bodies with broad-spectrum fluorescence emission^36^ are conspicuous (Fig. 2g, h), and not found in young (see Supplementary Fig. 2), These autofluorescent inclusions, which represent classic hallmarks of aging^37,38^, initially presented an obstacle to morphometric analysis. However, we noted that by using 405 nm excitation with a 570–610 nm collection window, we could independently image autofluorescence, and thus remove it from reporter images (documented in Supplementary Fig. 2). Confetti labeling in aged cTEC was conspicuously less robust than that seen in young (Fig. 2i vs. 2a). In many regions it was difficult to detect cTEC cell processes without ultra high magnification, but they do remain pervasive throughout the atrophied cortex in this diminished condition. Changes in cTEC cell morphology are most pronounced in the sub-capsular space (Fig. 2i), which notably represents the region where the highest density of age-associated pigments is seen (Fig. 2h). Various morphological parameters, such as total surface area, feret (maximum) diameter, or occupied cell volume (Fig. 2k–m) quantitatively confirm contraction of cTEC projections with age. In this context, we wish to emphasize that our measurements must underestimate the real difference, since the worst affected cTEC were so difficult to distinguish from background that they could not be measured. Nonetheless, quantitative, as well as qualitative findings show that aging is associated with a very significant diminution in cTEC morphology, represented by a contraction in projection length/complexity as well diameter.

To determine whether thymic regeneration correlated with rejuvenation of cTEC morphology, we analyzed aged Confetti thymuses 21 days after surgical castration, representing peak regenerated size^21^. In general, cTEC morphology at this time was greatly enhanced, although the cortex was not identical to young (Fig. 2j vs. 2a)^21^, despite similar size. Instead, cTEC morphology in the regenerated thymus was widely variable, with the conspicuous appearance of regions of unusually large cTEC, as well as other regions where cTEC morphology remained poor, qualitatively (Fig. 2j) or quantitatively (Fig. 2k–m). The reason for this non-uniformity is difficult to pin down, but might reflect cells that were more or less damaged by aging. Again, our quantitative analysis (Fig. 2k–m) probably underestimates the frequency of very large cells, since they are more likely to exceed the boundaries of any fixed optical volume, and thus cannot be measured. Consequently, the quantitative measurements shown in Fig. 2k–m are conservative, but nonetheless confirm re-extension after regeneration. It is worth noting that age associated autofluorescent bodies remain a prominent feature in the fully regenerated thymus (see Supplementary Fig. 2), further emphasizing our previous finding that even though cellularity of the thymus may be temporarily restored by castration, the underlying consequences of aging are permanent^21^.

### mTEC morphology is unchanged during atrophy and regeneration

**Fig. 3:**
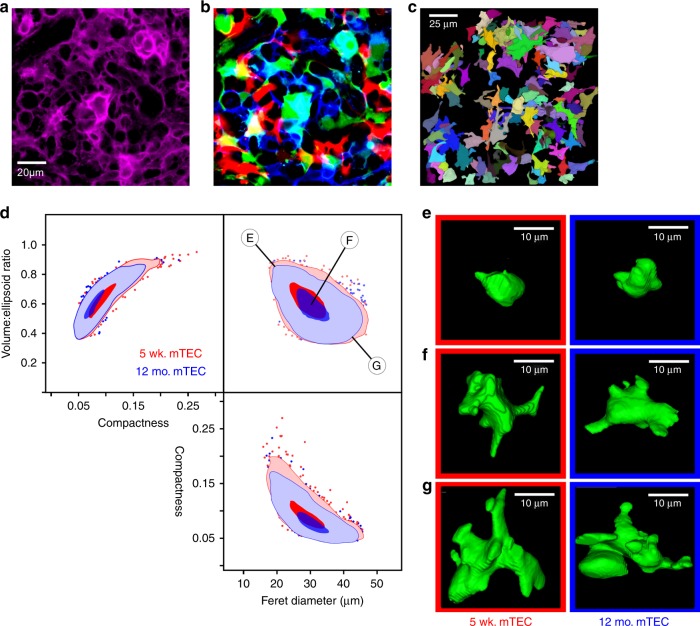
mTEC exhibit a broad spectrum of shapes that change little during atrophy. **a** A maximum Z projection (2.5 µm thickness) of medulla from a young (5 weeks) Confetti thymus, stained with a conventional mTEC marker (Epcam). Note that individual cells cannot be distinguished. **b** Confetti fluorescent proteins (mCfp, cYfp, and cRfp) in the same tissue volume, showing clear distinction of individual cells and their shapes. Scale bar in **a** also applies to **b**. **c** 3D projection of a 25 µm-thick stack of one color channel (cYfp) from the young Confetti medulla, with individual cells labeled by a random palette. **d** Orthogonal projections of the 3D distribution of mTEC shapes from young (red) or old (blue) mice. At least 1000 objects are shown for each age. Dark colors indicate 10% probability limits, light colors show 90% probability limits, and the remaining events are shown as individual dots. An overlapping (between young and old) unimodal distribution is found, but with a broad diversity of shapes (**e**–**g**), ranging from larger and extensively branched to smaller and non-branched morphologies

Our previous work showed that atrophy and regeneration are primarily functions of changes in cTEC, while the medulla is minimally affected^21^, consistent with the findings of others^30–32^. However, it is possible that there could be qualitative changes in mTEC that do not result in changes in medullary volume. In any case, like cTEC, the morphology of individual mTEC is poorly defined. To accomplish this, we used the cYfp and cRfp choices in Confetti, which produce filled objects (cells) that can be readily segmented and characterized. To define ideal parameters defining individual mTEC, we generated mice carrying one *Rosa26[Confetti]* allele and one *Rosa26* allele bearing a conditionally stopped histone 2b (H2b):mCherry fusion protein^39^, resulting in red fluorescent labeling of all TEC nuclei after induction of both alleles by Foxn1[Cre]. This allowed us to train the segmentation approach (see Methods) until there was reasonable certainty (>95%) that we were defining individual mTEC containing a single nucleus. This approach was then applied to cYfp and cRfp image stacks from both young and aged Confetti mice. As shown in Fig. 3 and Supplementary Movies 4–6, Confetti revealed a diverse range of mTEC morphologies, in contrast to cTEC, which had essentially a single canonical shape. These diverse mTEC morphologies did not fall into identifiable subtypes, but rather formed a continuous distribution (Fig. 3d–g), even when other morphological parameters were simultaneously tested for clustering. Using three highly discriminatory parameters (feret diameter, the largest possible linear distance between two points of an object; compactness, the ratio of the surface area of an object to its volume; or volume:ellipsoid ratio, the ratio of the volume of an object to the volume of the smallest circumscribing ellipsoid), the shape distributions of young and aged mTEC were virtually indistinguishable (Fig. 3d), further emphasizing that the primary target of accelerated age atrophy in the thymus is cTEC and the cortex, while mTEC and the medulla remain essentially undisturbed.

### TEC numbers remain stable during atrophy and regeneration

**Fig. 4:**
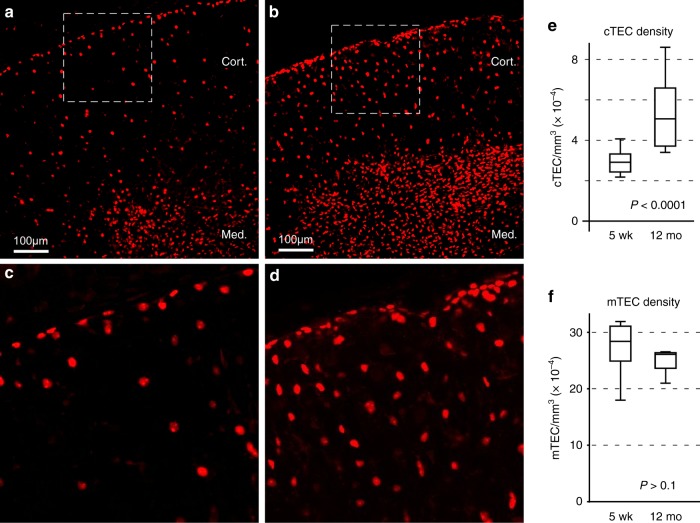
cTEC density increases with atrophy, indicating contraction of cell size without changing in number. **a**, **b** Wide area view of thymus from 5 week or 12-month-old mice (respectively) in which an H2b:mCherry fusion protein is conditionally activated in all cells of the TEC lineage. Images represent a maximum Z projection of an optical slice with thickness equal to the average feret diameter of one TEC nuclei (~11 µm). Paired images were acquired and processed identically. Regions indicated by dashed lines are shown in panels **c** and **d**. Consistent with this visual assessment and the contraction of cTEC projections by the Confetti reporter, measurement of TEC density shows that cTEC density increases dramatically with age, while mTEC density is not significantly changed (**e** and **f**, respectively; statistical significance calculated by two-tailed *t* test). Data indicate total nuclei per image volume from at least 3 independent thymuses of each type, with each thymus represented by one or more distinct image volumes until large numbers of nuclei were counted. Total events counted were 5060 (young cortex), 3567 (aged cortex), 17,097 (young medulla), or 10,176 (aged medulla). Boxes represent the interquartile range with the median indicated by a horizontal line; whiskers indicate the full range. Source data are provided in the accompanying Source Data File

Our lab and others have shown that thymic atrophy is primarily a function of decreased cortical volume^21,30–32^ resulting from age related changes in cTEC^21^. Confetti labeling (Fig. 2) shows that aging involves contraction of the extensive cell projections that characterize cTEC, and thus of overall cTEC size. Diminished size of individual cTEC, in the absence of cell death, should lead to an increase in the apparent density of cTEC. To test this, we used the *Rosa26[H2b:mCherry]* allele described above^39^, activated by Foxn1[Cre], to unambiguously label the nucleus of all TEC. Results are summarized in Fig. 4. Consistent with our predictions, aging resulted in a very significant increase in the density of TEC in the cortex, but not the medulla, both visually (Fig. 4a–d) and quantitatively (Fig. 4e, f). Consistent with a dominant role for the outer cortex in atrophy, the highest density of cTEC is seen in the outer cortex and sub-capsular region. Note that the capsule and the immediate sub-capsular region were actually excluded from quantitative density measurements, since their distribution approximates a curved 2D area density rather than a 3D volume density. Thus, the quantitative results presented for cortical density with age (Fig. 4) again underestimate the true extent of this process. Together with Confetti marking, these cell number/cell density experiments confirm that age related atrophy of the thymus is explained by contraction of cortical volume due to shrinkage of cTEC in the absence of cell death, resulting in an apparent increase in their density. Our findings further indicate that cortical atrophy initiates in the outer cortex/subcapsular region (also coincident with the highest density of age-associated pigment inclusions), and gradually progresses inward.

Relevant to our estimates of TEC number, a recent study used a combination of conventional staining and reporter labeling to quantitate TEC in the young thymus^40^, and concluded that mechanical/enzymatic methods for isolating TEC from the thymus grossly underestimate the total number of TEC found in situ. They conclude that many TEC either are not efficiently released by these methods, or they are destroyed in the process. Our results strongly support this, and the genetic labeling approach we used, which has the advantage of labeling only TEC while ignoring everything else, suggests that the number of TEC is even higher. Our measured densities (Fig. 4) applied to the tissue volumes estimated by those authors suggest that cTEC number in the young thymus is about 3-fold higher than their measurements (9.9 × 10^5^ cTEC per lobe, vs. 3.7 × 10^5^), and 5-fold higher for mTEC (2.6 × 10^6^ total, vs. 4.9 × 10^5^). Our findings are very consistent with an earlier electron microscopy study^41^, which showed 6.8 × 10^5^ cTEC and 2.6 × 10^6^ mTEC per thymus at 4 weeks of age. Regardless of any differences between these studies, all of them reinforce the conclusion that TEC are much more numerous than predicted by the yields from physical isolation. This suggests that methods for removing TEC from the thymus yield a minor proportion of the total cells, which may or may not be representative of the remainder or the group as a whole. Not surprisingly, for reasons related to their complex and fragile morphology, this discrepancy is greatest among cTEC, which are apparently isolated with a very low success rate (see Discussion).

### Regeneration does not require changes in TEC proliferation

To this point, our data support the conclusion that age-related atrophy of the cortex results from contraction of cTEC projection morphology, rather than by senescence-associated death of cTEC. The corollary is that regeneration can be explained primarily by the re-extension of cTEC cell projections, independent of proliferation. Several laboratories have addressed proliferation in mTEC using in vivo DNA labeling^42–44^, but always by analyzing isolated cells, which, as described above, is now known to dramatically underestimate the total population, and may be skewed towards certain cell types. As a further complication, labeling was always performed for multiple hours to days, which can result in production of labeled non-mitotic daughter cells (i.e., one mitotic precursor producing two post-mitotic progeny), thus obscuring the true proliferative index. Thus, a clear static assessment of mTEC proliferation in situ is lacking. Among cTEC, the situation is even less clear. One study suggested that castration restored the mTEC/cTEC ratio^45^, but data were not shown. Another study^46^ indicated that IL-22 can increase cTEC proliferation, but basal levels in untreated mice were again not shown, and mechanically isolated cells were used. Thus, the proliferative status of cTEC in the young, aged, and regenerated thymus remains unresolved.

**Fig. 5:**
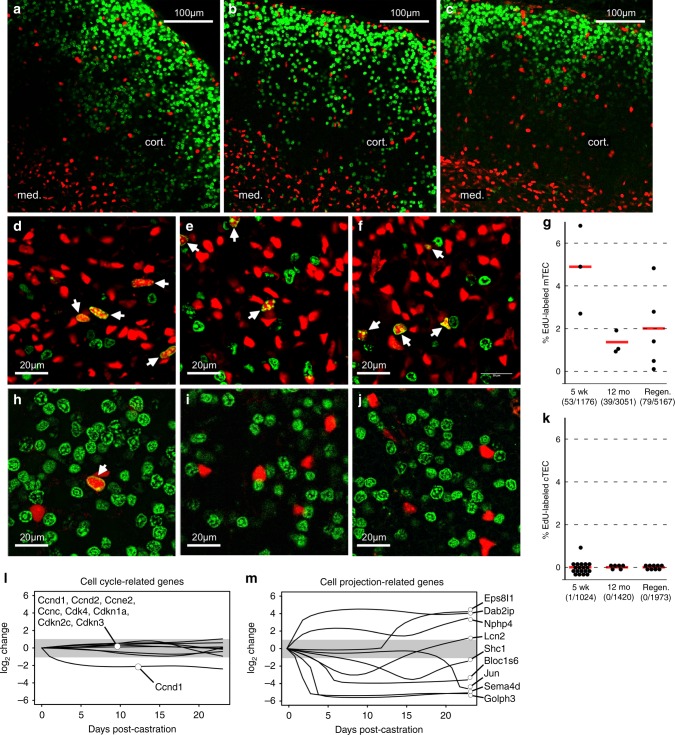
Regeneration of the cortex occurs independently of cTEC proliferation. **a** Wide area view of a single optical plane of thymus from a 5-week-old mouse in which H2b:mCherry (red) was conditionally activated in TEC by Foxn1[Cre], 1 h after a single injection of the thymidine analog 5-ethnyl-2′-deoxyuridine (EdU, green). EdU^+^ nuclei are not obvious in the deeper tissue because display levels are limited by the intense staining of cortical lymphoblasts, but they are nonetheless found throughout the organ. **b**, **c** Similar wide area views from 12 month thymuses, or thymuses from 12-month mice at the midpoint of regrowth (day 14 post-castration), respectively. **d**–**f** Higher magnification views of the medulla in thymus from 5 weeks, 12 months, or regeneration day 14. Edu^+^ mTEC nuclei are readily detectable (arrows) in all tissues, although the frequency does decrease with age (**g**), a feature that does not change after regeneration. Data points represent the percentage of labeled nuclei in individual image volumes from three biologically independent thymuses for each sample type (young, aged, regenerated). Red bars indicate the mean of these percentages. Numbers below sample types indicate pooled EdU^+^ nuclei/pooled total nuclei for each sample type. **h**–**j** Similar analysis of the cortex in the same order of appearance. A single Edu^+^ cTEC was found out of 1024 nuclei that were formally counted in multiple volumes from at least 3 tissues) (**h**, **k**); many more volumes were examined (but not formally counted) without finding additional labeled cells. No labeled nuclei were found in cTEC from 12 months or regenerating thymus showing that tissue regeneration does not correlate with an increase in cTEC proliferation. **l** Consistent with the near absence EdU labeling in cTEC, genes associated with cell cycle do not fluctuate during castration-induced regeneration; only one of these (cyclin b1) exhibits a 2-fold or greater change (indicated in gray) during induced regrowth. **m** For comparison, an equal number of dynamically regulated genes found within the cell projection ontologies (see Fig. 1). Source data are provided in the accompanying Source Data File

To address this, we used the thymidine analog 5-ethnyl-2′-deoxyuridine (EdU) to efficiently label cells in S phase in *Foxn1[Cre]/H2b:mCherry* mice in an acute (60 min) in vivo pulse, followed by copper-catalyzed detection^47^ using a fluorescently tagged alkyne (ThermoFisher # C10337). Thymus sections were taken from young (5-week-old) mice, as well as from 12-month-old mice prior to castration or at mid-phase of the regeneration response (day 13 most-castration, see Fig. 1a and (day 13 post-castration, see Fig. 1a and^21^), where proliferation of any cell should be present at a maximal rate. Intensely proliferating lymphocyte progenitors conveniently served as an internal control for EdU penetrance, and were brightly labeled throughout the thymus (Fig. 5a–c), with very high density in the outer cortex, as previously described^8,48^. Labeled mTEC were readily detected at all ages (Fig. 5d–f). In the young thymus, approximately 4.5% of all mTEC were in S phase (Fig. 5g), which, in general, is consistent with values obtained using different methods and labeling times^44,45^. Also consistent with previously published work^45^, we find that proliferation of mTEC is reduced approximately threefold with age (Fig. 5g). However, even with a large number of unambiguously defined mTEC being counted, we found no change in mTEC proliferation during regeneration (Fig. 5g), consistent with a minimal role for the medulla in this process^21^.

In contrast to mTEC, changes in cTEC are a dominant factor in age related atrophy and regeneration (Figs. 2 and 4, and ref. ^21^). Consequently, if proliferation of cTEC were a major mechanism for regeneration of the thymus after castration, one would expect to see a conspicuous increase in EdU-labeled cTEC after castration. However, as shown in Fig. 5h–k, this was not the case. Proliferating cTEC were very rare or non-existent in the cortex, even in the young thymus, where a single EdU-labeled cTEC was found among dozens of image volumes representing thousands of cTEC (note: the image volumes described for Fig. 5 were formally quantitated, but many more were manually examined without formal measurement). In the aged or regenerating thymus, no labeled cTEC could be found at all. It might be tempting to contemplate a technical error, except that lymphocyte labeling in the same regions is conspicuous (Fig. 5h–j, green only), and unlabeled (by EdU) cTEC nuclei are also readily visible (red only). Consequently, we conclude that proliferation among cTEC is very limited in the steady-state young thymus, and does not increase during regrowth. Consistent with EdU labeling, genes associated with cell cycle do not change significantly during castration induced regrowth (Fig. 5l), with the exception of a modest change in cyclin B1, which may be playing a non-cell cycle related role, or may be spurious, but in any case cannot act alone to promote cell division. Together, data from Confetti and EdU labeling experiments confirm that the non-presumptive predictions made by our temporal gene expression database (Fig. 1) are correct, and that cortical atrophy and regeneration are controlled by modulating the extensive cell projections found in young cTEC, rather than a change in cTEC number.

### Trans-acting medullary signals may regulate cTEC morphology

**Fig. 6:**
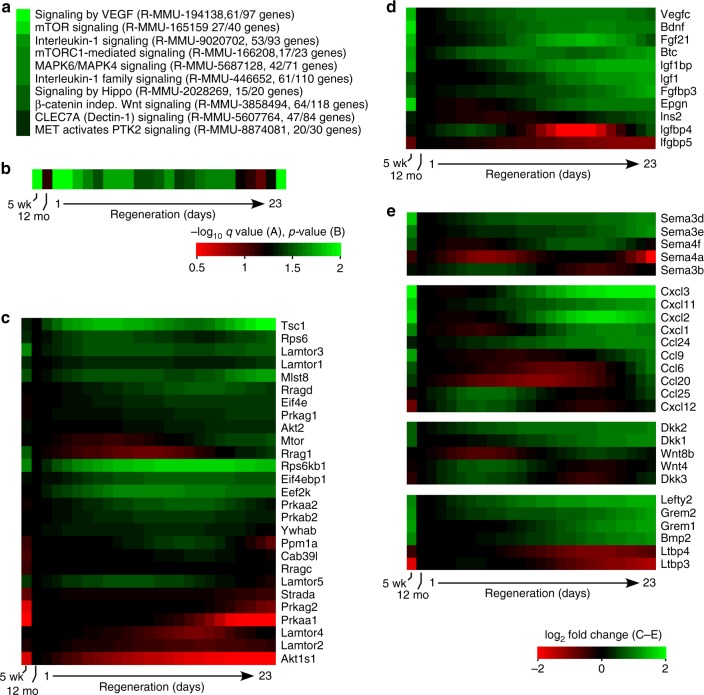
Pathways regulating cell size or shape suggest control of cTEC morphology by medullary ligands. **a**–**c** Representation of data from cortical stroma, while **d**, **e** derive from medullary stroma. **c**–**e** Normalized to the 12-month-old untreated thymus, which also represents day 0 of the regeneration sequence. **a** The top 10 Reactome signaling pathways (sorted by hypergeometric *q* value) mapping to genes expressed by cortical stroma. Reactome pathway designations, and the number of genes expressed in thymic stromal cells vs. the total genes in the pathway, are shown in parentheses. Note that 3 of the top 4 pathways impact mTor signaling, making mTor a prime candidate for changes in cTEC morphology during aging and regeneration. **b** Change in representation (determined by the Fisher exact test) of the mTor pathway in cortical stroma during atrophy and regeneration. **c** Dynamics of mTor pathway genes in cortical stroma during atrophy and regeneration. **d** Dynamic regulation of mTor-activating ligands by medullary stroma; particularly notable are Igf1 and Fgf21. **e** Medullary stroma also dynamically regulate other classical paracrine mediators of cell growth and morphology, including members of the Sema, chemokine, Wnt, and Tgfb signaling pathways. Source data are provided in Supplementary Data 1

Our database of stromal and lymphoid gene expression during aging and regeneration allowed us to predict that the size and shape of cTEC, rather than changes in their number, were the driving force behind atrophy and regrowth. While regulation of cell size and shape are highly complex and multidimensional processes not amenable to rapid dissection, the same database, together with existing knowledge of critical regulators of cell size and shape in many developing organisms, allows us to make mechanistic predictions about this process for further study. We used the enrichPathway function of the ReactomePA package in R to define Reactome pathways (reactome.org) that were significantly enriched in cortical stroma (see Methods). These were then limited to pathways with ≤200 genes associated with the keywords signaling or signaling (spelling variants). The ten most significant of these pathways are shown in Fig. 6a; 8 of these 10 (excluding *IL1* and *Clec7A*) have obvious strong ties to cell size, shape, and epithelial remodeling. Note that 3 of the 4 most significant pathways are associated with Mtor, which is widely recognized as a critical regulator of cell and tissue size via stimulation of energy metabolism, protein and lipid synthesis, and cytoskeletal reorganization^49,50^. Notably, Mtor is also recognized as a high priority target for aging intervention and increased longevity^51^, and inhibition of Mtor activity (via treatment with rapamycin) has been shown to diminish thymus size^52^. Further strengthening these associations, the significance of the Mtor pathway in cortical stroma changes dramatically with aging and regeneration (Fig. 6b), with relatively high representation in the young or during regrowth, and diminished representation in the aged or in terminal regeneration. Analysis of the individual Mtor pathway genes expressed by cortical stroma also reveals dynamic regulation of many pathway components (Fig. 6c), including the key regulatory component Tsc1.

Soluble ligands targeting the Mtor pathway, and more broadly, targeting most classical pathways associated with cell size and shape (e.g., *Wnt*, *Tgfb*, etc), were generally not expressed or were not dynamically regulated by cortical stroma. Many of these could possibly be endocrine in origin, although it is important to remember that the thymic cortex (but not the medulla) is immunologically privileged, and is protected by a nearly impermeable blood-thymus barrier^53^. However, we find that medullary stroma is enriched in, and dynamically regulates, multiple ligands for Mtor and other morphogenic pathways (Fig. 6d), including recognizable TEC signals such as Igf1^54^ or Fgf21^55^. Our data thus suggest that multiple pathways regulating cell size and shape in other tissues also control the branching morphology of cTEC, with a dominant role for Mtor. Further, it appears that induction of these pathways in cTEC may be under the dynamic control of paracrine factors produced by medullary stroma, which is apparently responding to systemic endocrine signals induced by castration. Overall, our findings are most consistent with a mechanism in which cortical atrophy represents progressive degeneration of cTEC morphology due to diminished production of paracrine signals from the medulla, while castration-induced regeneration represents a transient replenishment of these signals, and the corresponding restoral of cTEC morphology and cortical volume.

## Discussion

Since T cells must be continuously produced throughout life, the accelerated atrophy of the thymus with age is enigmatic, especially given its latent regenerative potential. Age-associated atrophy is common to many tissues (for instance, muscle, CNS, skin, and testes), and can be a consequence of cell loss (death), but often is associated with the shrinkage (atrophy) of individual cells, collectively resulting in tissue atrophy. Likewise, regeneration may be attributed to proliferation of stem or end stage cells (hyperplasia), but can also result from growth of existing cells (hypertrophy) that is independent of proliferation. Despite the magnitude of atrophy and regeneration in the thymus, the underlying mechanisms for these processes have remained obscure, although our previous findings show that both are attributable to changes in cTEC^21^. Our non-presumptive analysis of global gene expression in young cTEC, aged cTEC, or aged cTEC in the regenerating thymus suggested that genes associated with cell size and shape dominated the dynamic landscape (Fig. 1). However, the size and shape of individual cTEC has been difficult to discern using conventional methods, thus obscuring any changes that might occur during age atrophy or regeneration.

Here, we used Confetti conditional labeling to accurately define the size and shape of individual TEC, as well as H2b:mCherry conditional labeling to unambiguously define TEC number and density without obstruction by other cell types. Our results reveal novel aspects of TEC biology (especially among cTEC) that broadly influence our interpretation of their normal function. The morphology of cTEC is absolutely unique, as far as we can tell, in all of metazoan biology. The closest cellular correlate may be Purkinje cells, but unlike these and other truly branched cells, which are characterized by serial bifurcations that eventually terminate, cTEC cell projections consist of labyrinthine, looping structures forming about 20–30 small cavities per cTEC, each of which contains about 2–10 lymphoid cells on average (e.g., about 150 lymphoid cells per cTEC). All cTEC from young thymus (other than those lining the capsule or blood vessels) are very similar in overall size and shape (defined in Fig. 2d and Supplementary Movie 2), represented by a slightly flattened ovoid with a long axis of approximately 75 μm, with the long axis generally perpendicular to the capsule. Based on this unimodal size, it appears that the overall dimensions of cTEC are specified genetically (within the constraints of the local microenvironment), much like any other cell type. In contrast, the organization of looping cell projections within cTEC is highly variable, and thus seems unlikely to be genetically specified. Instead, it appears these complex projections are defined and constrained by the shape of the lymphocytes that they contain. We believe that this shaping of cTEC projection morphology by lymphoid cells illustrates the well-recognized phenomenon of crosstalk^7^, where each cell type requires the presence of the other for normal development. The intimate wrapping of lymphocytes by cTEC projections, together with their unimodal size and shape, compels us to reflect on the potential relationship of cTEC to thymic nurse cells^56^, which are defined as a rare population of cTEC that completely enclose a variable number of immature lymphocytes. We believe that the apparent rarity may be explained by the very small proportion of cTEC expected to survive mechanical disruption of the tissue, based on the complex and fragile morphology we show here. While cTEC defined by Confetti do not completely engulf their associated lymphocytes, it is worth noting that this particular characteristic of nurse cells was demonstrated in vitro, not in situ^56^. Consequently, we now conclude that all cTEC essentially represent nurse cells or, stated another way, that nurse cells are no different than the average cTEC. In the context of cTEC acting as the matrix for lymphoid progenitor migration (reviewed in ref. ^4^), we also note that the morphology of cTEC defined by Confetti suggests that lymphoid progenitors migrate through individual cTEC, rather than upon them, perhaps an esoteric distinction, but one that is consistent with the nurse cell concept of engulfment of lymphoid cells.

Defining the morphology of cTEC (and mTEC) allowed us to test the predictions of our aging/regeneration database (Fig. 1), which suggested that changes in cell projection morphology were dominant. Confetti labeling in 12-month-old mice showed that cortical atrophy is indeed characterized by contraction of cTEC cell projections, while regeneration correlates with their re-extension. We note that neither contraction during aging nor regrowth during regeneration is uniform among all cTEC. In atrophy, cTEC contraction is most conspicuous in the outer regions of the cortex (Fig. 2g, i, and Supplementary Movies 1 and 2), resulting in a disproportionate increase in cTEC density in this area (Fig. 4). This same region corresponds to the site of intense lymphoid proliferative activity (Fig. 5, and refs. ^8,48^), and thus, of intense anabolic metabolism. Notably, the autofluorescent inclusions that are hallmarks of metabolic damage during aging^37,38^ are also concentrated here (Fig. 2h and Supplementary Fig. 2e, f). We have previously shown that cTEC express low levels of the antioxidant enzyme catalase, and thus they rapidly accumulate oxidative damage^57^. Therefore, it makes sense that this process would be most conspicuous in the outer cortex, where intense anabolic metabolism occurs as the consequence of rapid lymphoblastic proliferation (Fig. 5, and refs. ^8,48^). From all these observations, we conclude that cortical atrophy (and, therefore, thymic atrophy) results from a toxic cycle of anabolic lymphoid metabolism, cTEC susceptibility to the byproducts of that metabolism, accumulated metabolic damage in cTEC, and their resultant atrophy, a process that initiates in the sub-capsular cortex and progressively moves inward through the cortex with age.

Like degeneration with age, regeneration among cTEC after castration is also non-homogenous. Although the overall appearance of the cortex (as defined by Confetti) is dramatically improved (Fig. 2j), it is associated with significant hypertrophy in some cells, but little change in others. We are unable to predict whether heterogeneity among cTEC responses during regeneration reflects cells with differing levels of accumulated damage, or whether it is simply stochastic, although the former seems the more intuitive option. In any case, the regeneration that does occur is sufficient to fully reconstitute the cortex (and thus, the thymus), albeit transiently^21^. We believe that the transient nature of thymic regeneration^21^ is more consistent with cTEC growth, rather than proliferation, as a mechanism; cTEC generated de novo from stem/progenitor cells would be expected to have a relatively normal lifespan, and to degenerate much more slowly, instead of returning to the atrophied state within 2 weeks^21^. In contrast, changes in cell projection morphology can be easily accommodated by a 2-week time frame. Our overall conclusion from analysis of dynamic gene expression (Fig. 1), cell morphology (Fig. 2), cell density (Fig. 4), and cell proliferation (Fig. 5) is that thymic atrophy and regeneration both reflect modulation of cTEC morphology (size), which occurs independently of appreciable levels of cell death or proliferation. We wish to point out that we refer here only to the accelerated atrophy of the thymus seen at a relatively young age (for instance, the 75% reduction in cellularity seen by 12 months of age in mice, and proportionally in all other species with a thymus). It is possible, if not likely, that senescence-associated cell death plays an increasing role at much later age, as it does in other tissues.

In contrast to the relatively unimodal gross size and shape found in young cTEC, mTEC display a wide diversity of sizes shapes that do not cluster into identifiable groups, but instead represent a broad, continuous spectrum (Fig. 3). It is unclear whether or not extremes in this distribution are functionally or genetically distinct. However, it is interesting to contemplate that in the cortex, where cTEC are all essentially identical, lymphoid progenitor cells follow a synchronous path involving proliferation, commitment to the T lineage, TCR/MHC screening, etc. In contrast, in the medulla, where mTEC morphology varies widely, multiple T lineages with distinct functional capacities diverge (helper, cytotoxic, regulatory, etc). Thus, it seems worth considering that mTEC heterogeneity may reflect distinct roles in specifying different T cell functions, a hypothesis that is readily testable using Confetti-labeled mTEC and various T lineage markers.

In addition to visualizing the distinct morphologies of individual TEC, our studies also reveal a previously unrecognized role for medullary signals in the maintenance and regulation of cTEC morphology. While classical response pathways determining cell size and shape (most notably, Mtor) are dynamically regulated by cTEC themselves during atrophy and regeneration (Fig. 6), the inductive ligands are generally not produced in that compartment, either by stromal or lymphoid cells. This class of ligands can be of endocrine origin, but it is important to note that the thymic cortex is immunologically privileged, protected by a blood vessel barrier that tightly restricts entry of exogenous substances^53^. The medulla, in contrast, is quite permeant to exogenous molecules^53^, and thus is much more likely to be exposed to endocrine or other systemic cues. We find that a number of ligands relevant to pathways that change in cTEC during atrophy and regeneration are produced by medullary stroma, and are dynamically regulated during aging and regeneration (Fig. 6). This implies the existence of an endocrine/paracrine signaling axis, were the medulla responds to peripheral (endocrine) cues by signaling to the cortex in paracrine fashion, at least one consequence of which is modulation of cTEC morphology. Dependence of the cortex on the presence of a medulla was first noted more than 50 years ago^58^, although the nature of this dependence has still not been elucidated. Our data suggests that it may be explained, at least in part, by dependence of cTEC on morphogenic signals derived from medullary stroma, either in the steady state or during atrophy/regeneration. How exogenous substances entering the thymus via the medulla are subsequently prevented from entering the cortex^53^, while intrinsic medullary products (for instance, the chemokines CCL21 and CCL25^59^) can readily do so is completely unclear. In this respect, it is interesting to note that a formal barrier between medulla and cortex has never been defined, even though simple histological analysis indicates that it must exist, since the lymphoid and stromal contents of these compartments do not mix. Regardless of how this barrier is formed, our findings suggest that the cortical response seen after castration does not result directly from androgen signaling to cTEC, but rather reflects responses by medullary stroma to the endocrine environment (possibly including androgen signaling), subsequently resulting in regulation of cTEC morphology. Our data also suggest that even though mTEC morphology and density (Figs. 3 and 4) and the size of the medullary compartment^21^ do not change much during aging, the ability of medullary stroma to produce signals controlling cTEC morphology does diminish with age, in a manner that is transiently restored^21^ by the systemic response to castration.

## Methods

### Mice

C57Bl/6 mice (JAX strain 000664), *Foxn1[Cre]* knockin mice (JAX strain 018448^35^), *Rosa[Confetti]* mice (JAX strain 013731^34^), or *Rosa[H2b:mCherry]* mice (JAX strain 023139^39^) were purchased from Jackson Laboratories, and bred or crossed at Scripps. All experimental mice were male. For surgical castration, animals were anesthetized with ketamine (120 mg/kg) and xylazine (16 mg/kg) administered intraperitoneally. The surgical site was sterilized by swabbing with Betadine and then ethanol, and a 3–4 mm incision was made through the skin and tunica surrounding testicle using a scalpel. The testes were exposed and all associated vessels were clamped with a hemostat, followed by ligation using 4–0 silk sutures, and excision of the testes. The site was then closed using Vetbond adhesive (3 M, St. Paul, MN, USA). The procedure was repeated individually for each testicle. Animals were given 0.2 ml of sterile saline subcutaneously, placed in a sternal recumbent position on a heating pad, and monitored until they became ambulatory. Buprenorphine (0.05 mg/kg) was administered to relieve pain immediately after the mice became ambulatory, and every 8 h thereafter until activity had returned to normal. All animal use was compliant with ethical standards, and was reviewed and approved by the Scripps Institutional Animal Care and Use Committee.

### Cell, tissue, and RNA isolation

These procedures were performed using our previously validated methods^21,60^. Microdissected tissue and lymphoid cells were paired, in that tissue was isolated from one lobe of the thymus, and lymphoid cells from the other. For tissue isolation, intact thymus lobes were removed and immediately placed in ice cold OCT mounting medium, then frozen. Transverse 20 µm sections were cut from the middle third of the organ (A/P axis) and mounted on PEN membrane slides (Leica). Sections selected for microdissection were chosen to have cortical regions 400–600 μm deep, medullary regions at least 200 μm deep, and symmetrical cortical/medullary borders. Tissue was fixed in cold acetone/ethanol (3:1 v/v), rehydrated through graded ethanol, stained with cresyl violet (LCM Staining Kit, Ambion), and dehydrated through graded ethanol. Slides were dried at room temperature and immediately used for microdissection on a Leica AS LMD system. Sections adjacent to those used for microdissection were mounted on glass slides for archival purposes. Additional sections 100 μm above and below microdissected sections were also mounted on glass slides and examined to rule out tissue irregularities outside microdissected plane. Cortical subregions were prepared from tissues as described above, with each subregion representing 15% of the total cortical depth. RNA was prepared using RNAqueous Micro kits (Ambion). Each independent RNA pool included tissue from multiple sections and mice.

For lymphoid cells, thymus lobes were removed and placed immediately in ice cold medium (Hanks’ balanced salt solution, 5% FBS, 10 μg/ml DNAse), with all subsequent steps at 4 °C. Single cell suspensions were stained with labeled antibodies (below), followed by cell sorting. Sort gates for medullary lymphoid cells were CD3^hi^ AND CD45^+^, and for cortical lymphoid cells were CD3^-/lo^ AND CD45^+^ AND (CD90^+^ OR CD117^+^). Biotinylated CD3 antibody was from eBioscience, catalog # 13-0031-86, lot # E 0234801631 used at 1:50 dilution, followed by Alexa 647-streptavidin from Molecular Probes, catalog number S-11229, lot # 65A1-1, used at 2 μg / ml. PE-conjugated CD45 antibody was from Caltag, catalog # RM6404, lot # 01020803, used at 1:100 dilution. Alexa-647-conjugated CD90 antibody was from BioLegend, catalog # 105318, lot # B175617, used at 1:25 dilution. FITC-conjugated CD117 was from eBioscience, catalog # 11-1171, lot # E00475-330, used at 1:200 dilution. Dead cells were excluded using DAPI. Cells for microarray analysis were selected to be >99% pure, and were generally >99.8% pure. RNA was prepared using RNeasy Mini Kits (Qiagen) according to recommended protocols.

### In vitro transcription, RNA labeling, and array hybridization

These were carried out by the Genomics Core Facility at The Scripps-Florida Research Institute. Each RNA template consisted of at least three pooled RNA samples of the same type (e.g., cortical lymphocytes) but from different thymuses, in order to minimize sample variation. 0.2–1.0 µg of total RNA was used for a single cycle of in vitro transcription. Biotin-labeled cRNA probes were then prepared as recommended by the manufacturer (Affymetrix) using oligo-dT primers, SuperScript, and BioArray High Yield RNA Transcript kits (Affymetrix). Fragmentation/hybridization to MOE430v2.0 arrays (Affymetrix) were performed according to standard Affymetrix protocols. Imaging was performed on a Hewlett Packard GeneArray Scanner. MIAME-compliant raw data has been deposited into the NCBI Gene Expression Omnibus database (GSE132136). The total raw data includes 156 arrays. Each data point includes 4 microarrays, representing lymphoid cells or microdissected tissue from cortex or medulla. Twenty serial time points are included, including 5-week-old mice, untreated 12-month-old mice (day 0 of regeneration), or castrated mice at each day from day 6 to day 23 after surgery. At least one array was generated for each sample type (cortical or medullary, lymphoid cells or tissue) on each day, with additional replicates at key inflection points: young (5 independent arrays per cell/tissue type), day 0 (4 independent arrays per cell type), day 6 (3 independent arrays per cell type), day 7 (2 independent arrays per cell type), day 12 (3 independent arrays per cell type), day 16 (2 independent arrays per cell type), day 17 (3 independent arrays per cell type), and day 20 independent arrays per cell type).

### Identification of genes expressed by stromal cells

All processing was performed in R^61^. All 156 .cel files were normalized using the rma() function from the R package affy^62^, with the normalize option set to TRUE. A permissive (minimum of false negatives) list of genes designated as being expressed in the thymus (stromal or lymphoid) was first defined as those genes in tissue microarrays that received a majority of non-absent detection calls (i.e., Present or Marginal, using the mas5calls() function in the R package affy) at any time point where 3 or more independent replicates were present, or, where the signal value was above the chip median in the majority at any time point where 3 or more independent replicates were present. For each probeset in this list, 3rd degree polynomial splines were fit to the regeneration time-course for each sample type (days 0 to 23 post-castration) using the ns (R base package splines), rlm (R package MASS^63^), and predict (base package stats) functions, with the hampel method for outlier detection and down weighting. Internal knots were placed at days 6 and 17. This splined (smoothed) data for all expressed genes (tissue or lymphoid) was used for subsequent processing. Stromal gene expression was calculated using our previously published approach^60^, using the formula stromal expression = [tissue signal–(lymphoid signal × lymphoid proportion)]/(1−lymphoid proportion), where tissue signal is the measured (splined) tissue signal values, lymphoid signal is the measured (splined) lymphoid signal values, and lymphoid proportion is the average ratio of lymphoid to tissue signal values for a manually curated list of known lymphoid-specific genes^60^. The resulting calculated stromal values were then quantile renormalized to the corresponding spline fit data for each day. When data from young thymuses were included in the analysis, stromal signals were calculated similarly but using measured tissue and lymphoid values in place of spline-fit values. For analyses where a minimum of false positive stromal genes was desirable (for instance, Fig. 1), at the expense of more false negatives, a high confidence stromal gene list was defined by setting a minimum tissue:lymphoid ratio threshold equal to the mean + 2.5 s.d. for the lymphoid-specific list of genes. For analyses where a minimum of false negatives was desirable (for instance, Fig. 6), at the expense of more false positives, this threshold was set to mean + 2.0 s.d. Additional details specific to Figs. 1 or 6, but not both, are provided in the corresponding Results sections.

### Enrichment analysis

Gene Ontology enrichments were performed using the hyperGTest() function from the R package GOstats^64^ with the conditional option set to TRUE, and with ontology mappings from the org.Mm.eg.db package^65^. Reactome enrichments were performed using the enrichPathway function from the ReactomePA package in R^66^, with all genes as background. Overrepresentation was calculated using the fisherexact function in the base R package stats.

### Isolation and preparation of tissue for microscopic imaging

After euthanasia, the thymus was removed and placed immediately into ice-cold 2–4% formaldehyde (Polysciences #18814) in PBS, followed by gentle rotation at 4 °C for 6–16 h. Graded sucrose infiltration (10/20/30% in PBS) was then performed for ~24 h at 4 °C, followed by cryogenic mounting in OCT. Tissues were sectioned to the appropriate thickness (depending on the application, 5 μm to 150 μm) and mounted on positively charged glass microscope slides. Tissues were never allowed to dry, even momentarily. OCT was removed by incubation at room temperature for 1 h in PBS, followed by washing in PBS, mounting, and coverslipping. For tissue >10 μm thick, mylar spacers of an appropriate thickness were used between the slide and coverslip to prevent compression of the tissue.

### Confocal microscopy

Imaging was performed using a Leica SP5 Resonant Scanner (housed at the Max Planck Florida Institute, Jupiter, FL) equipped with 405 nm, 458 nm, 561 nm, and 633 nm diode lasers, as well as an argon ion laser producing several spectral lines (most relevant, 488 nm and 514 nm). For virtually all studies shown here, a 20× glycerol, long working distance immersion objective was used, and 8-bit gray scale images were obtained.

### Confetti image acquisition

The Confetti allele encodes three fluorescent proteins (membrane Cfp, cytoplasmic Yfp, and cytoplasmic Rfp) that are useful for defining the full extent of labeled cells, and a fourth (nuclear Gfp) that was of limited value to the present studies. To minimize the detection of nGfp while acquiring the other Confetti colors, and simultaneously including a channel for subtraction of autofluorescent age pigments, a three stage sequential scan was used. All three scans used identical collection windows of 463–509 nm (window 1), 519–556 nm (window 2), and 566–628 nm (window 3). In the first scan, 405 nm and 458 nm lasers were used simultaneously (since neither of them was optimal) to excite both mCfp and autofluorescent pigments, corresponding to collection windows 1 and 3, respectively. Note that autofluorescent pigments of age excite maximally in the UV/violet range but emit across a broad spectrum of wavelengths, and thus, window 3 contained exclusively autofluorescent activity. In the second scan, a 514 nm laser was used to maximally excite cYfp (and minimally, nGfp), which was collected in window 2 (again, biased in favor of cYfp). In the third scan, a 561 nm laser was used to excite cRfp, collected in window 3. For full color displays, mCfp is presented as blue, cYfp as green, and cRfp as red. When an additional immunofluorecent stain was added, the fluochrome was Alexa 633 or Alexa 647 excited by the 633 nm laser, and a fourth detection window of 638nm-700nm was collected simultaneously with cYfp in the second stage scan, since Yfp and these far red dyes were spectrally non-overlapping.

### Confetti cTEC image analysis

All image analysis in this study was performed using ImageJ software^67,68^ with associated plugins, as noted. Morphological analysis of cTEC was a particular challenge, due to their large size (and thus, frequent truncation by the volume boundaries), the small diameter of their extensive cell projections, and extensive degeneration with age. The following is a general description, although some small adjustments may have been applied non-identically, as necessary, as described below. Individual cells (defined by any single color surrounded by cells of other colors) contained wholly within the image volume were identified, and a corresponding smaller volume containing the entire cell was cropped out. Contrast enhancement (Process > Enhance Contrast) was applied (0–3% saturation, as appropriate for any given cell), and a 3D median filter (Process > Filters > Median 3D, *x*.*y* radii = 2, *z* radius = 1) was applied. Segmentation was performed (Plugins > boneJ > Optimize Threshold)^69^ to generate a binary image, and surrounding cells or parts of cells were removed (Plugins > boneJ > Purify). In some cases, additional noise removal (Process > Noise > Remove Outliers, or Process > Noise > Despeckle) or background subtraction (Process > Subtract Background) may have been applied to generate a clean image for quantitation. Cell parameters, including volume, surface area, feret diameter, etc, were then calculated (Plugins > 3D > 3D Manager > Measure 3D) using 3D manager^70^.

### Confetti mTEC image analysis

The first step in this process was to use tissue from (young) mice carrying one *Rosa26* Confetti allele and one *Rosa26* H2b:mCherry allele (as well as *Foxn1[Cre]*) to validate the conditions by which individual mTEC could be distinguished within the tightly packed medullary compartment. The approach was refined by manual evaluation of H2b:mCherry overlaid onto the results, until it was clear (using a subset of images) that the approach was generally identifying objects that contained one, and only one, nucleus. For this analysis, only the cYfp channel was used to identify mTEC, because cRfp was in the same image channel as mCherry (separable by morphology), and mCfp resulted in a hollow image that was not readily amenable to counting. The following defines the process, which varied little to none between images. Process > Enhance Contrast (1% saturation), background subtraction (Process > Subtract Background, rolling ball radius = 10, sliding paraboloid), and median 3D filtering (Process > Filters > Median 3D, *x*,*y* radii = 2, *z* radius = 1) were applied to further enhance the Yfp image and distinguish it from background or noise. A copy of this file was made for watershedding, as described later. 3D minimum filtering (Process > Filters > Minimum 3D, *x*,*y* radii = 2, *z* radius = 1) was applied to facilitate separation into individual objects. Next, the Plugins > 3D > 3D Edge and Symmetry filter was applied (alpha Canney = 0.3, radius = 10, normalization = 10, scaling = 2, with improved seed detection) to define individual objects (cells). The resulting symmetry smoothed file was restored to 8-bit pixel depth, and brightness levels were enhanced (min = 5, max = 45). Plugins > 3D > 3d Maximum Finder (*x*,*y* radii = 25, *z* radius = 10, noise = 75) was then used to assign a single peak to each object, for use as a seed for watershedding. The image copy described above was smoothed (Plugins > Process > Smooth 3D, Gaussian value = 0.5), and the watershed was applied to this image with the peaks as seeds, seeds threshold = 50, and image threshold = 20. Analyze > 3D > 3D Objects Counter (threshold and size minima = 1, exclude on edges, with objects boxes) was applied and the resulting image was restored to pixel density and size. 3D parameters were generated using Plugins > 3D > 3D Manager > Measure 3D with default parameters. These measurements, together with H2b:mCherry co-expression (above), were used to define filters to remove objects (cells) containing no nucleus or more than one nucleus. For the analysis presented in Fig. 3, object volume was constrained such that the lower threshold was greater than the average volume of an mTEC nucleus plus 3σ (i.e., > 532 μm^3^), while the upper threshold was set to exclude objects in the top 5% of three manually selected parameters (feret diameter, compactness, and volume:ellipsoid ratio). For Fig. 3, kernel density estimates were generated using the kde() function from the R package ks^71^.

### Image analysis for measurement of TEC density

Raw images generally consisted of 0.25 × 0.25 × 0.1 mm confocal image stacks. In order to facilitate automated counting, image stacks were processed using a 3D median filter (radii = 2), rolling ball radius background subtraction (radius = 25), and noise reduction (Process > Noise > Remove outliers, with radius = 5 and threshold = 1). The processed stack was then thresholded (Process > Adjust > Threshold, minimum = 10, maximum = 255), the resulting stack was inverted, and then subtracted from the original image. Noise reduction (above) was then carried out once more to remove any small objects (noise) prior to application of the 3D maximum finder^70^ with *xy* radius = 10, *z* radius = 3, noise = 10, to define a point representing the fluorescence maximum of each nucleus. Accuracy was manually evaluated for each stack by overlaying a colorized version of the fluorescence maximum point onto the original H2b:mCherry image. Final quantitation represents multiple distinct tissue volumes from at least 3 independent thymuses in each anatomic compartment at each age. Note that capsule proximal volumes were excluded from cortical counting, due to the 3D curvature of the organ.

### In vivo metabolic labeling of DNA

One hour prior to euthanasia, mice received a single intraperitoneal injection of 5-ethnyl-2′-deoxyuridine (ThermoFisher A10044) at a dose equimolar to that previously described^8^ for BrdU (40 μg/g body weight). Tissue was then fixed in formaldehyde and processed in graded sucrose for cryosectioning, as described above. Tissue sections (50 µm thick) were permeabilized for 1 h at room temperature in PBS containing 3% BSA and 0.5% Tween-20, followed by copper catalyzed click labeling with an Alexa 488 azide conjugate (ThermoFisher #C10337) for 45′ at room temperature, several washes in PBS/BSA, and confocal imaging.

### Reporting summary

Further information on research design is available in the Nature Research Reporting Summary linked to this article.

## Supplementary information

Supplementary Information

Reporting Summary

Description of Additional Supplementary Files

Supplementary Data 1

Supplementary Movie 1

Supplementary Movie 2

Supplementary Movie 3

Supplementary Movie 4

Supplementary Movie 5

Supplementary Movie 6

Source Data

## Data Availability

The datasets generated during and analyzed during the current study are hosted by GEO GSE132136. The source data underlying Figs. 2k–m, 3d, 4e–f, and 5g, k, l, and m are provided in the Source Data file.
