# Supplementary material for: Dynamic changes in epithelial cell morphology control thymic organ size during atrophy and regeneration
Source: Nat Commun. 2019 Sep 27;10:4402. doi: 10.1038/s41467-019-11879-2 (PMC6765001; doi:10.1038/s41467-019-11879-2)
Supplement: Supplementary file 1 — Supplementary Information [file 41467_2019_11879_MOESM1_ESM.pdf]

Supplemental Information

Dynamic changes in epithelial cell morphology control thymic organ size during atrophy and regeneration.

Venables et al.

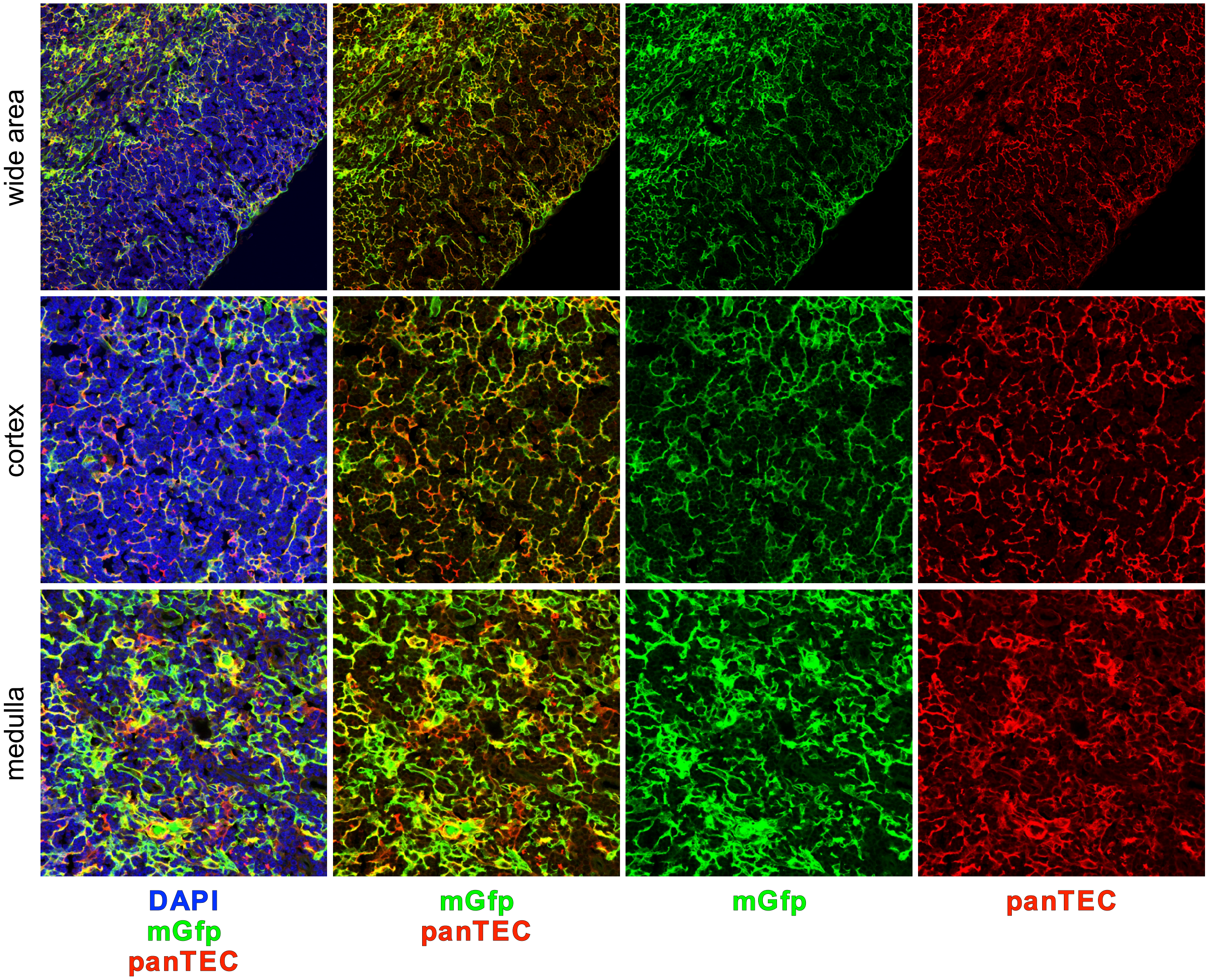

Supplemental Figure 1. Foxn1[Cre] activates Rosa26-driven conditional reporters in virtually all TEC. A basic premise of this study is that Foxn1[Cre] activates conditional reporter alleles in all TEC. This seems intuitive, since all but a tiny fraction of TEC are known to express Foxn1 and/or derive from a Foxn1-expressing precursor. However, as a formal proof, tissue sections (5µm) were taken from the thymus of a 5 week-old mouse carrying a conditional allele nearly identical to those used in the main text (i.e., Rosa26[floxSTOPflox-reporter]), but in which a membrane-targeted Gfp is homogenously expressed upon activation by Foxn1[Cre] (Muzumdar et al., PMID 17868096). Sections were also stained standard fashion (see Methods) using a panel of antibodies recognizing a wide range of consensus mTEC or cTEC markers (panTEC), including Epcam, Ly-51, Dec205, Krt5, and Krt8, all visualized using Alexa 647. All cells staining with the pool of consensus TEC markers also expressed Gfp, and additional cells that did not stain (or stained at low levels) with TEC markers were also Gfp labeled. These findings show that the Foxn1[Cre] strain used in our studies does efficiently activate Rosa26-driven conditional reporter alleles in essentially all TEC. Antibodies or secondary tags were as follows: anti-Epcam-biotin, eBioscience catalog # 13-1591-82, used at 1:100 dultion; Alexa 647-streptavidin, Invitrogen catalog # 521374, 1:200; purified anti-Krt5, Biolegend catalog # 905501, 1:200 dilution; Alexa-647-conjugated goat-anti-rabbit IgG, Jackson Immunoresearch catalog # 11-606-03, 1:200 dilution; anti-Krt8 supernatant, Developmental Studies Hybridoma Database catalog # AB-531826, 1:500 dilution; Alexa-647-conjugated mouse-anti-rat IgG, Jackson Immunoresearch catalog # 212-606-168; Alexa-647-conjugated anti-Ly51, BioLegend catalog # 108311, 1:50 dilution; Alexa-647-conjugated anti-DEC205, eBioscience catalog # 138203, 1:50 dilution.

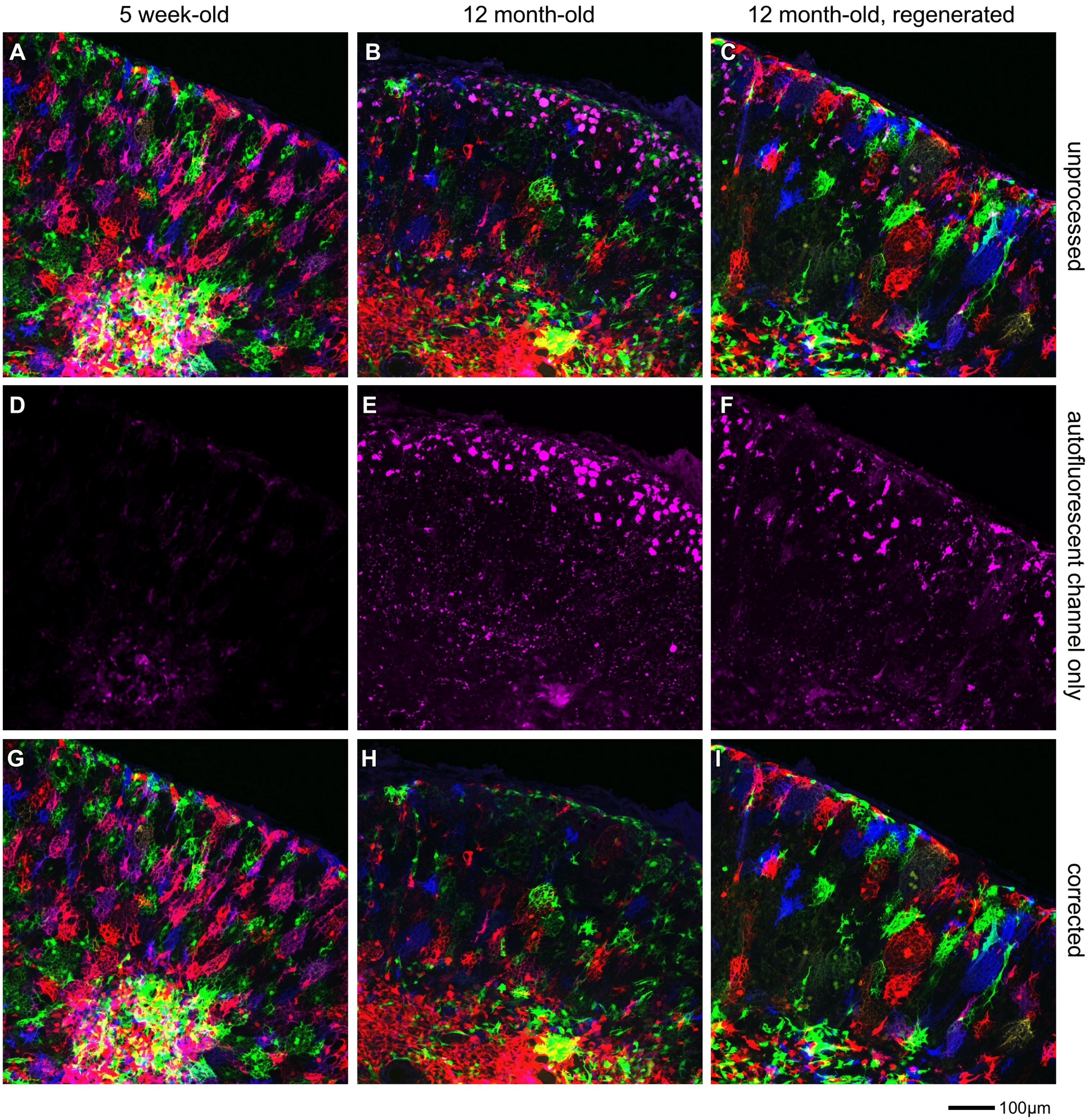

Supplemental Figure 2. Correction of raw images for removal of autofluorescent pignments that accumulate with age. A-C represent maximum z projections of a 40µm-thick optical section (approximately one cTEC thick in the non-radial plane) from Foxn1[Cre] Confetti thymus from different types of mice, as indicated. D-F represent fluorescence collected in a 566nm-628nm (red) window after stimulation with a violet (405) laser. Note that minimal autofluorescence is seen in the young thymus, but high levels are seen in aged and regenerated thymus, with highest density in the sub-capsular cortex (aged) or outer cortex (regenerated). G-I represent the original images (A-C) after subtraction of this autofluorescence. Note that little change is induced in the legitimate Confetti colors. G-I are identical to the images presented in A, I, and J (respectively) of Figure 2.
