# Supplementary material for: Dynamic changes in epithelial cell morphology control thymic organ size during atrophy and regeneration
Source: Nat Commun. 2019 Sep 27;10:4402. doi: 10.1038/s41467-019-11879-2 (PMC6765001; doi:10.1038/s41467-019-11879-2)
Supplement: Supplementary file 3 — Description of Additional Supplementary Files [file 41467_2019_11879_MOESM3_ESM.pdf]

Description of Additional Supplementary Files

Supplementary Data 1 consists of an Excel file displaying deconvolved stromal signal values for a list of high confidence stromal genes. See Methods for additional details. The median signal value for the chip was ~45. Cortical stroma and medullary stroma are on separate tabs. Note that regeneration day 0 of regeneration also represents unmanipulated 12 month-old stromal data.

Supplemental Movie 1 (related to Figure 2A,I,J). An animated axial projection through 50 μm of thymus tissue from Foxn1[Cre] Rosa[Confetti] mice at 5 weeks of age, 12 months of age, or 21 days after castration of 12 month old mice. Blue = Cfp; green = Yfp; red = Rfp.

Supplemental Movie 2 (related to Figure 2D). A animated 3D rotation of a single cTEC showing looping projections and the corresponding intracellular voids. This is the canonical morphology defined by Confetti, represented by a slightly flattened ovoid approximately 75 μm in the long dimension.

Supplemental Movie 3 (related to Figure 2F). An animated 3D rotation of a high magnification view of the interface between two distinct Confetti-labeled cTEC, showing that connection of cells in the cTEC matrix occurs mainly via abutment of looped cell projections, rather than terminal synapses.

Supplemental Movie 4 (related to Figure 3C). An animated 3D rotation of a single fluorescent protein channel (cYfp) from a 100 μm-thick optical slice of 5 week-old Confetti medulla. After segmentation as described in the Methods, individual mTEC were assigned unique colors to distinguish them from one another.

Supplemental Movie 5 (related to Figure 3D). An animated 3D rotation of the morphometric distribution of mTEC in young vs. aged thymus. Axis represent probability distributions for three morphometric parameters that would be expected to reliably distinguish most existing shapes: feret diameter, the largest possible linear distance between two points of an object; compactness, the ratio of the surface area of an object to its volume; or volume:ellipsoid ratio, the ratio of the volume of an object to the volume of the smallest circumscribed ellipsoid. Darker shading represents the central 10% of the population, lighter shading represents the central 90%, and dots represent the remaining events.

Supplemental Movie 6 (related to Figure 3E-G). Animated 3D views of individual mTEC at the two extremes or the center of shape distributions shown in Figure 3D or Suppl. Figure 7. The locations of these cells within the distributions are indicated in Figure 3D.
